# High efficiency hybrid white organic light-emitting diodes based on a simple and efficient exciton regulation emissive layer structure†

**DOI:** 10.1039/c8ra08753a

**Published:** 2018-12-06

**Authors:** Yuwen Chen, Shian Ying, Qian Sun, Yanfeng Dai, Xianfeng Qiao, Dezhi Yang, Jiangshan Chen, Dongge Ma

**Affiliations:** Institute of Polymer Optoelectronic Materials and Devices, State Key Laboratory of Luminescent Materials and Devices, South China University of Technology Guangzhou 510640 People's Republic of China msdgma@scut.edu.cn

## Abstract

It is well-known that hybrid white organic light-emitting diodes (WOLEDs) are constructed by blue fluorophors and red-green or orange phosphors, therefore, theoretically exhibiting the advantages of long lifetime and high efficiency. However, the efficiency is far from reaching the expected values. Here, we designed a simple and efficient exciton regulation emissive layer (EML) structure to fabricate high efficiency hybrid WOLEDs. The EML sequentially comprises a yellow EML of phosphor doped hole-transporting material, a blue EML of a blend of hole- and electron-transporting materials and an exciton regulation layer of ultra-thin green phosphor inserted in electron-transporting material. It can be seen that the emissive excitons are well regulated by such a strategic EML structure. The resulting WOLEDs exhibit a maximum forward viewing external quantum efficiency and power efficiency of 18.2% and 72.9 lm W^−1^, respectively, and they are as high as 16.7% and 61.7 lm W^−1^ at 100 cd m^−2^, and 12.5% and 37.7 lm W^−1^ at 1000 cd m^−2^, showing the positive properties of high efficiency and low efficiency roll-off. Such outstanding performance will greatly promote the development of high performance hybrid WOLEDs.

## Abstract 2

High performance hybrid WOLEDs based on a blue fluorescent EML and inserted an exciton regulation layer of ultrathin green phosphor.

## Graphical abstract

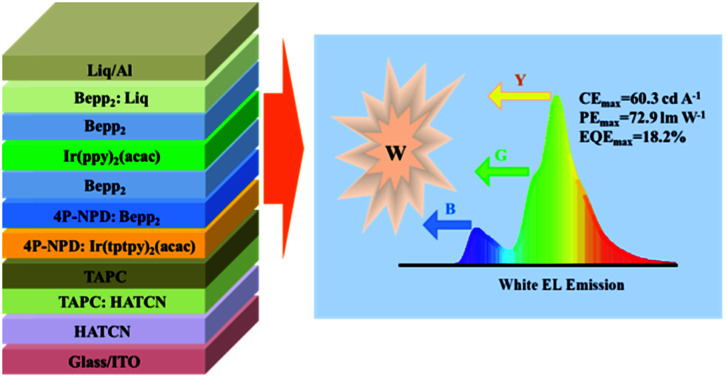

## 1. Introduction

It is known that organic light-emitting diodes (OLEDs) have been the subject of a great deal of research since C. W. Tang demonstrated the first OLED in 1987^1^ and J. Kido reported the first white OLED (WOLED) in 1994.^2^ Academia and industry have been devoted to the improvement of electroluminescence (EL) efficiency and long term stability of fabricated OLEDs. WOLEDs have been known to be an ideal lighting source because of the advantages of no shadows, soft light, no blue light damage, large area, flexibility and transparency. Therefore, it is urgent to further improve the efficiency and stability of WOLEDs in order to satisfy the requirement of practical application in lighting fields.

Currently, three types of WOLEDs are reported according to the employed emissive materials, including all-phosphorescent WOLEDs, all-fluorescent WOLEDs and fluorescent/phosphore-scent hybrid WOLEDs.^3^ To enhance the efficiency, the utilization of phosphors is desirable since they can allow a conversion of up to 100% of injected charges into emitted photons, resulting in a theoretical internal quantum efficiency of unity.^4^ Unfortunately, there are no ideal blue phosphorescent emitters in terms of lifetime and stability up to now. This restricts the development of all-phosphorescent WOLEDs, which is a major challenge.^5^ As we know, conventional blue fluorophors show good stability, therefore, the hybrid WOLEDs with stable blue fluorescent emitters combining high efficiency long wavelength phosphorescent emitters become the best choice. Hybrid WOLEDs can be achieved by incorporating either two complementary colours (blue and yellow or orange) or three primary colours (blue, green and red) in two architectures, including single-emissive-layer (single-EML) structures^6,7^ or multi-emissive-layers (multi-EML) structures.^8^ However, as we see, the efficiencies of all reported hybrid WOLEDs are basically less than 60 lm W^−1^, and the efficiency roll-off at high luminance is also very severe.^9–11^

In this work, we fabricated high efficiency hybrid WOLEDs by strategically designing a simple and efficient exciton regulation emissive layer (EML) structure. It can be seen that not only the efficiency, but also the efficiency roll-off at high luminance are improved by the efficient control of exciton recombination zone in the blue fluorescent EML and the regulation role of the inserted ultrathin green phosphorescent layer in the excitons. The resulting hybrid WOLEDs shows a maximum external quantum efficiency and power efficiency of 18.2% and 72.9 lm W^−1^, and they are as high as 16.7% and 61.7 lm W^−1^ at 100 cd m^−2^, and 12.5% and 37.7 lm W^−1^ at 1000 cd m^−2^, respectively.

## 2. Experimental methods

### 2.1 Materials information

Dipyrazino[2,3-*f*:2′,3′-*h*]quinoxaline-2,3,6,7,10,11-hexacarbonitrile (HAT-CN) as the hole injection material, *N*,*N*′-di-(1-naphthalenyl)-*N*,*N*′-diphenyl-[1,1′:4′,1′′:4′′,1′′′-quaterphenyl]-4,4′′′-diamine (4P-NPD) as the yellow phosphorescent dopant host and blue emitter, di-[4-(*N*,*N*-ditolyl-amino)-phenyl]cyclohexane (TAPC) as the hole-transporting material, and bis[2-(2-hydroxyphenyl)-pyridine]beryllium (Bepp_2_) as the blue-emitting electron transport material were purchased from Jilin OLED Material Tech Co. Ltd. The yellow phosphorescent dopant iridium(iii)bis(4-(4-*t*-butylphenyl)thieno[3,2-*c*]pyridinato-*N*,C2′)acetylacet-onate (Ir(tptpy)_2_(acac)) and green phosphorescent dopant bis(2-phenylpyridinato-*N*,C2)iridium(acetylacetonate) (Ir(ppy)_2_(acac)) were purchased from Luminescence Technology. All materials were used without further purification. The chemical structures of all organic materials and the blue OLED device structure are shown in Fig. S1.†

### 2.2 Device fabrication

All devices were fabricated on glass substrates pre-coated by a 180 nm thick layer of indium tin oxide (ITO) with a sheet resistance of 10 Ω per square. The ITO glass substrates were cleaned sequentially by using detergent and deionized water before use. Prior to the thin-film deposition, the substrates were treated with oxygen plasma for 3 min to improve the work function after dried at 120 °C. Afterwards, the ITO glass substrates were loaded into a vacuum chamber for organic layer deposition by thermal evaporation under a base pressure of 5 × 10^−4^ Pa. The film thickness and deposition rates of the functional materials were monitored by a calibrated crystal quartz sensor. The deposition rates of organic layers, Liq, and Al were controlled at about 1 Å s^−1^, 0.1 Å s^−1^, and 4–6 Å s^−1^, respectively. For the case of doping or blending of two materials together, they were co-evaporated by respectively controlling their evaporation rate in two individual heaters at a certain ratio. The ultrathin layers were fabricated by very low evaporation rate of 0.01–0.02 Å s^−1^. The overlap between ITO anode and Al cathode was 4 mm × 4 mm as the active emissive area. Moreover, all organic films for PL test were prepared by vacuum evaporation on the quartz substrate.

### 2.3 Measurement

The current–luminance–voltage characteristics were measured by using a computer-controlled source meter (Keithley 2400) equipped with a light intensity meter LS-110 under ambient atmosphere without encapsulation. The EL spectra were measured by a Spectrascan PR650 spectrophotometer. The EQEs were calculated from the luminance, current density, and EL spectrum, assuming a Lambertian distribution. The photophysical properties, including UV/vis absorption, photoluminescence (PL), and excitation spectra were measured by a Shimadzu UV-2600 spectrophotometer, and an Edinburgh Instruments FLS 980 spectro-fluorometer. Additionally, the PL transient decay curves of the films were performed on Quantaurus-Tau fluorescence lifetime measurement system (C11367-03, Hamamatsu Photonics Co.). All the results of devices were measured in the forward-viewing direction and all the measurements were carried out under an ambient atmosphere without encapsulation.

## 3. Results and discussion

### 3.1 Structure and performance of blue OLEDs

To make high-performance hybrid WOLEDs, it is very important to first optimize the blue emission property.^12^ Therefore, choosing good blue fluorescent materials is considered. In our study, *N*,*N*′-di-1-naphthalenyl-*N*,*N*′-diphen-[1,1′:4′,1′′:4′′,1′′′-quaterphenyl]-4,4′′′-diamine (4P-NPD), a deep blue emitter with a dominant emission peak at 425 nm, high triplet energy level (2.3 eV) and high PLQY (92%) as well as bis[2-(2-hydroxyphenyl)-pyri-dine]beryllium (Bepp_2_), a deep blue emitter possessing a high fluorescent quantum yield of >80% and a high triplet energy level(2.6 eV)^13^ were selected to fabricate the efficient blue OLEDs.

As known to all, both 4P-NPD and Bepp_2_ with deep-blue fluorescent emitters are widely used in OLEDs.^14–16^ To demonstrate the viability of 4P-NPD and Bepp_2_ as blue emitters, we mixed 4P-NPD and Bepp_2_ to fabricate blue OLEDs by optimizing different blending ratios. The device structure is ITO/HAT-CN (15 nm)/TAPC : HAT-CN (4/2, 95 nm)/TAPC (40 nm)/4P-NPD : Bepp_2_ (*X*, 13 nm)/Bepp_2_ (5 nm)/Bepp_2_:Liq (4.5%, 50 nm)/Liq (1.25 nm)/Al (120 nm), where *X* is 1 : 0, 2 : 1, 1 : 1, and 1 : 2, corresponding to device B1, B2, B3 and B4, respectively. In these devices, considering that 4P-NPD shows good hole transporting capacity with a high mobility of 6.6 × 10^−4^ cm^2^ V^−1^ s^−1^,^8^ and Bepp_2_ exhibits excellent electron transporting capacity with a high mobility of 10^−4^ cm^2^ V^−1^ s^−1^,^17^ we use TAPC (di-[4-(*N*,*N*-ditolyl-amino)-phenyl]cyclohexane) with higher mobility (about 10^−2^ cm^2^ V^−1^ s^−1^) as the electron-blocking layer (EBL),^18^ and hole-transporting layer (HTL) in the blue devices. And TAPC has a low refractive index, a high T_1_ of 2.87 eV and a high LUMO (2.0 eV), which will well confine excitons and light coupling output.^19^ Fig. S1† illustrates the blue device structure. The chemical structures of all organic materials used in this study are given in Fig. S2.†

**Fig. 1:**
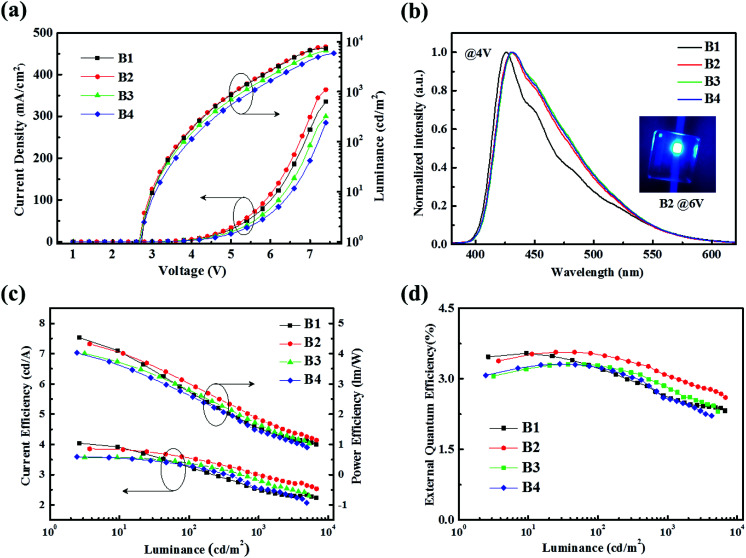
(a) Current density–luminance–voltage (*J*–*L*–*V*) characteristics of the resulting four blue OLEDs. (b) Normalized EL spectra of the resulting four blue OLEDs at 4 V. The inset shows the EL emission image of the blue device B2 at 6 V. (c) Current efficiency–power efficiency–luminance (CE–PE–L) characteristics of the resulting four blue OLEDs. (d) External quantum efficiency *versus* luminance (EQE-L) characteristics of the resulting four blue OLEDs.

Fig. 1(a) shows the current density–luminance–voltage (*J*–*L*–*V*) characteristics of the resulting blue OLEDs with different blending ratios. Clearly, they show the same turn-on voltage of 2.8 V, and the higher concentration of 4P-NPD in this blend leads to the higher luminance and bigger injection current density. The blue device with 2 : 1 concentration ratio emits the maximum luminance of 7880 cd m^−2^. Fig. 1(b) shows the normalized EL spectra of devices B1–B4 at a driving voltage of 4 V. It can be found that all devices emit blue light at a peak wavelength of about 427 with wide peak at long wavelength besides device B1. Obviously, the long wavelength emission should be from Bepp_2_ molecules. This proves the blue light in devices B2–B4 from the simultaneous emission of 4P-NPD and Bepp_2_ molecules.

Fig. 1(c) and (d) describe the current efficiency–power efficiency–luminance (CE–PE–L) and the EQE-luminance (EQE-L) characteristics of the four blue devices. The detailed EL performance parameters are summarized in Table 1. It's pretty obvious that the device B2 emits higher efficiencies, and also shows a lower efficiency roll-off at high luminance than the others under the same condition. The EQE of device B2 reaches the maximum value of 3.56% and keeps 3.03% at 1000 cd m^−2^ luminance, indicating the important role of the concentration ratio of 4P-NPD and Bepp_2_ blend. It can be seen that these devices also exhibit better pure blue emission with CIE (0.15, 0.12), which should be suitable for the preparation of WOLEDs.

**Table 1:** Summary of EL performances of the resulting four blue OLEDs

Device	*V*_on_ a (V)	*L*_(max)_ (cd m^−2^)	Max/@100 cd m^−2^/@1000 cd m^−2^			Peak^b^ (nm)	CIE^b^ (*x*,*y*)
			CE (cd A^−1^)	PE (lm W^−1^)	EQE (%)		
B1	2.8	7351	4.04/3.20/2.48	4.53/2.64/1.50	3.54/3.1/2.58	426	(0.16,0.12)
B2	2.8	7887	3.85/3.51/2.96	4.32/2.90/1.78	3.56/3.49/3.03	430	(0.15,0.12)
B3	2.8	6713	3.57/3.32/2.78	4.01/2.61/1.62	3.31/3.24/2.77	432	(0.16,0.12)
B4	2.8	5852	3.59/3.27/2.54	4.03/2.57/1.43	3.31/3.22/2.56	430	(0.16,0.12)

a Turn-on voltage estimated at a brightness of >1 cd m^−2^.

b Emission peak and the corresponding CIE coordinates obtained at a voltage of 4 V.

As we see, 4P-NPD and Bepp_2_ have the same highest occupied molecular orbital (HOMO) (5.70 eV for 4P-NPD and Bepp_2_) energy levels, whereas the small energy offset (∼0.3 eV) of lowest unoccupied molecular orbital (LUMO) (2.30 eV for 4P-NPD and 2.60 eV for Bepp_2_) energy levels.^16^ In addition, owing to the approximate carrier transporting abilities of 4P-NPD and Bepp_2_ and the good exciton confine property of TAPC, the main exciton generation zone should be located within the mixed blue emitting layer.

**Fig. 2:**
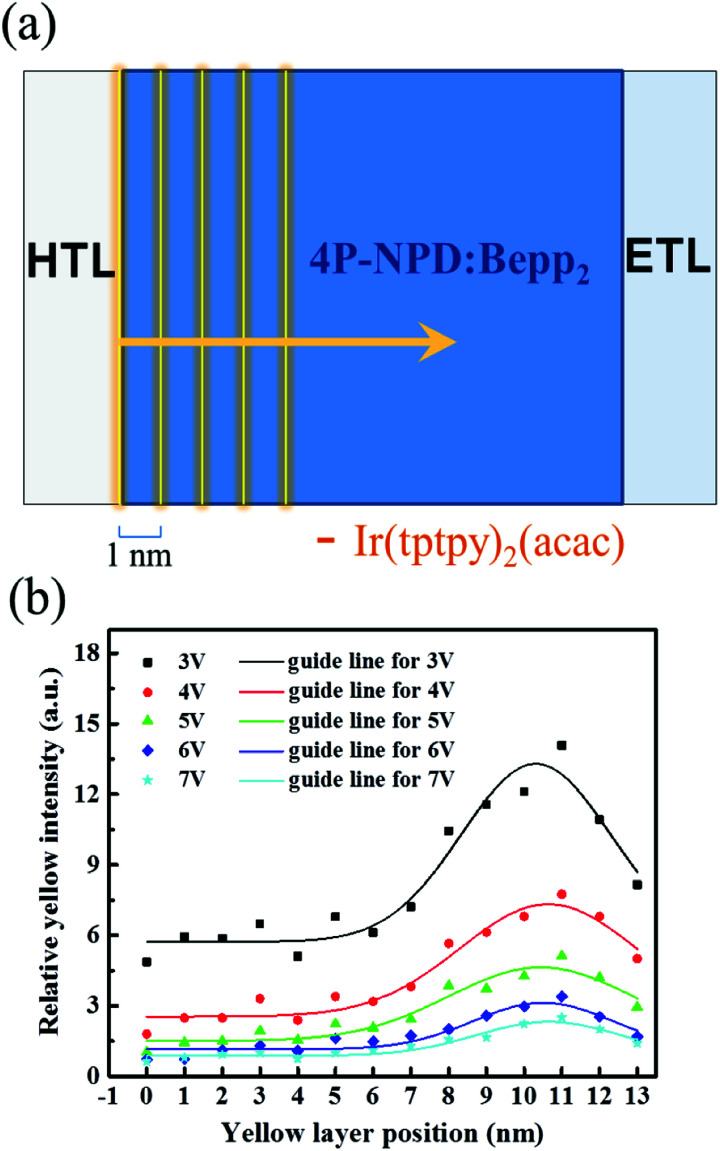
(a) Schematic diagram of the exploration on exciton distribution in the blue EML. (b) Ratio of yellow emission peak intensity to blue intensity as a function of the position of a yellow ultra-thin layer in the EML at a driving voltage of 3–7 V. The solid lines are the guides of the corresponding data.

Thus, we explored the exciton distribution profile in the 4P-NPD : Bepp_2_ blue EML by using the structure of ITO/HAT-CN (15 nm)/TAPC : HAT-CN (4/2, 95 nm)/TAPC (40 nm)/4P-NPD : Bepp_2_ (2 : 1, *X* nm)/Ir(tptpy)_2_(acac) (0.08 nm)/4P-NPD : Bepp_2_ (2 : 1, 13–*X* nm)/Bepp_2_ (5 nm)/Bepp_2_:Liq (4.5%, 50 nm)/Liq (1.25 nm)/Al, where *X* varies from 0 to 13 nm with an interval of 1 nm, as shown in Fig. 2(a), setting the interface at HTL/EML to be 0 position.

As shown in Fig. 2(b), the yellow emission is strongest at the position of 11 nm from the interface between HTL/EML, and remains higher intensity distribution in the range of regions from 7 to 13 nm. This implies that the exciton recombination zone in device B2 is close to the ETL side and its width is about 6 nm, guaranteeing the effective emission of blue light in devices based on 4P-NPD : Bepp_2_ as EML.

### 3.2 Structure and performance of hybrid WOLEDs

#### 3.2.1 Structure and emission processes of hybrid WOLEDs

**Fig. 3:**
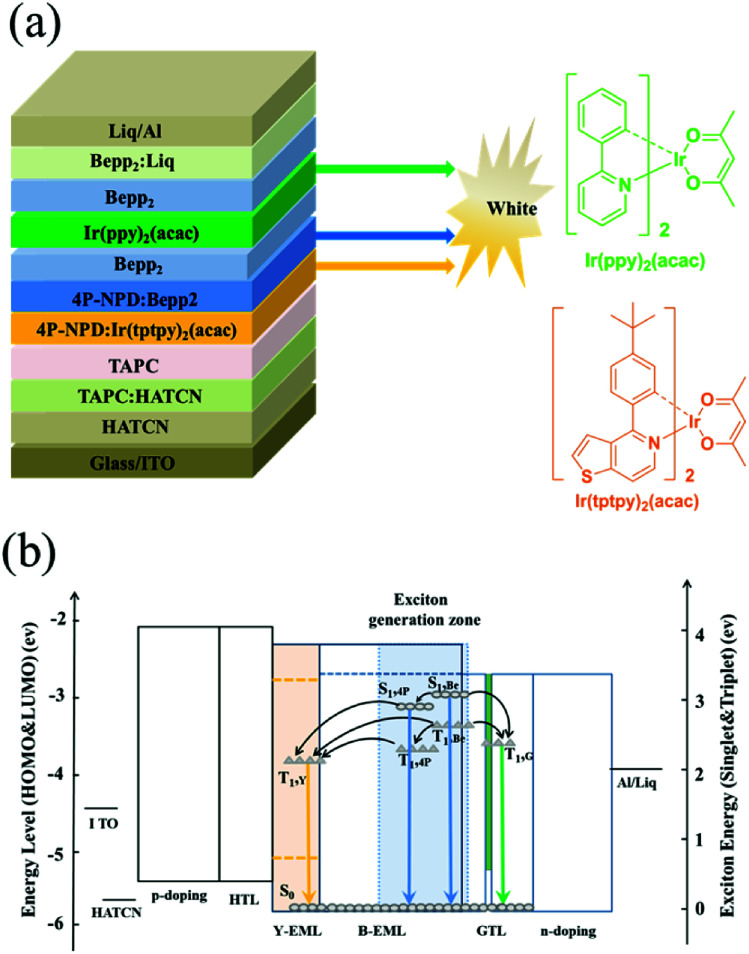
(a) Schematic structure diagram of the processed hybrid WOLEDs and chemical structures of the used phosphorescent materials. (b) Energy level scheme of the resulting hybrid WOLEDs, and exciton (S_0_, S_1_, and T_1_) energy diagram in EMLs. The gray filled rectangle represents the main exciton generation zone. Y, G, B, 4P and Be represents Ir(tptpy)_2_(acac), Ir(ppy)_2_(acac), 4P-NPD and Bepp_2_, respectively. The solid lines and dashed lines correspond to HOMO and LUMO energy levels, respectively. The circles and rhombus refer to the exciton (S_0_, S_1_, and T_1_) energies, respectively.

On the basis of the explored exciton distribution profile in the blue EML, and the motivation of design concept to achieve nearly 100% IQE, we fabricated hybrid WOLEDs (device W6) by adding a layer of doped yellow phosphor between HTL and blue EML and inserting an ultra-thin non-doped layer of green phosphor bis(2-phenylpyridinato-*N*,C2)iridium(acetylacetonate) (Ir(ppy)_2_(acac)) (0.4 nm) located at 2 nm from the interface of 4P-NPD : Bepp_2_/Bepp_2_. Apart from varying the EML structure, all other parameters of the devices are kept unchanged to allow for optimum comparability. The optimized white device W6 structure is as follows: ITO/HATCN (15 nm)/TAPC : HATCN (4/2, 95 nm)/TAPC (40 nm)/4P-NPD:Ir(tptpy)_2_(acac) (4%, 5 nm)/4P-NPD : Bepp_2_ (2 : 1, 13 nm)/Bepp_2_ (2 nm)/Ir(ppy)_2_(acac) (0.4 nm)/Bepp_2_ (3 nm)/Bepp_2_:Liq (4.5%, 50 nm)/Liq (1.25 nm)/Al. The schematic structure diagram of the processed hybrid WOLEDs and the chemical structures of the used phosphorescent materials are depicted in Fig. 3(a).

As given in Fig. 3(a), we rationally arranged the EML sequence to fabricate hybrid WOLEDs, thus enabled the device to achieve 100% IQE. In our proposed structure, as shown in Fig. 3(b), the exciton recombination region is strategically controlled in the blue EML, thus the singlet excitons produced from the exciton recombination region are mainly used for the blue light emission, and the triplets excitons transfer their energies to phosphorescent dyes for the yellow and green light emission.

Although 4P-NPD exhibits high PLQY, but its relatively low triplet state energy (2.3 eV) compared to Ir(ppy)_2_(acac) (2.4 eV) makes it not ideally located adjacent to Ir(ppy)_2_(acac) in hybrid WOLEDs.^14,20^ This problem can be solved very well by inserting an additional organic layer with high triplet level between them, which can prevent the energy transfer of the triplet excitons from Ir(ppy)_2_(acac) to 4P-NPD that causes unwanted energy loss. However, the use of the external interlayer has several disadvantages that limit the device quantum efficiency and power efficiency. First, the voltage drop across the interlayer is not negligible, leading to high voltage, thus low power efficiency. Furthermore, the interlayer maybe brings additional interfaces which inevitably increase the possibility of exciplex formation to some extent, impairing the efficiency of hybrid OLEDs.^8^ In our design, as shown in Fig. 3, we solved well this problem by simply introducing an ultra-thin green phosphor layer in Bepp_2_ ETL far from the blue EML without changing the device structure. Thus, the Bepp_2_ layer effectively suppresses the energy loss from Ir(ppy)_2_(acac) to 4P-NPD due to its higher triplet level (2.6 eV). Importantly, the ultra-thin green phosphor layer can well regulate the triplet excitons in the blue EML by energy transfer, thus reduces the quenching caused by exciton accumulation, but does not affect the electron transport in devices.^21–23^

From the relationship of the triplet energy levels shown in Fig. 3(b), it is obvious that the triplet exciton energies on 4P-NPD and Bepp_2_ can be well transferred to Ir(tptpy)_2_(acac), leading to the yellow emission. However, the singlet excitons in the blue EML can also directly transfer to Ir(tptpy)_2_(acac) molecules *via* Förster transfer, as a result, the blue emission in WOLEDs will be greatly reduced, leading to the white emission not balanced. Considering the fact that the singlet exciton has a shorter diffusion length,^24^ it provides a possibility to separate the singlet excitons spatially. As shown in Fig. 2(b) and 3(b), we effectively control the exciton recombination region in blue EML close to the ETL side, thus there is at least 5 nm separation distance to prevent the singlet excitons in blue EML from Ir(tptpy)_2_(acac) molecules, guaranteeing the enough blue emission. All in all, with such a working mechanism, all the generated excitons, both singlet and triplet excitons, can be fully utilized, realizing nearly 100% exciton harvesting while ensuring good white light emission.

#### 3.2.2 Performance of hybrid WOLEDs

**Fig. 4:**
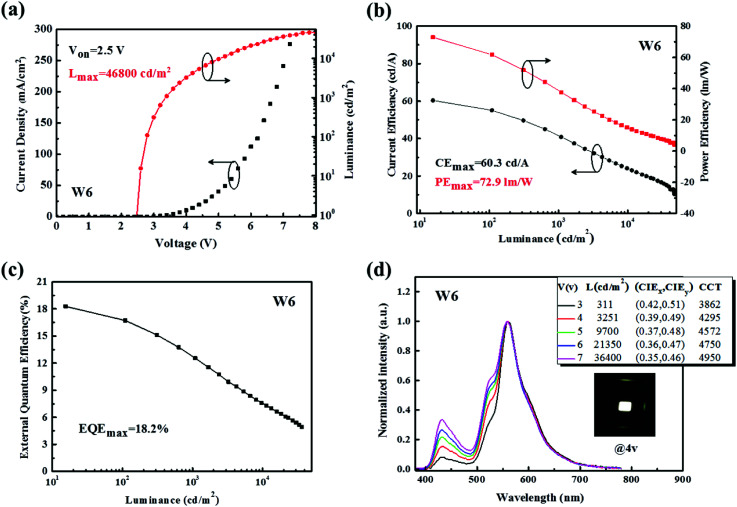
(a) *J*–*L*–*V* characteristics of the optimized device W6 with luminance measured in the forward direction without any out-coupling enhancement techniques. (b) CE–PE–L characteristics of device W6. (c) EQE-L characteristic of device W6. (d) Normalized EL spectra, luminance, CIE coordinates, and CCTs of device W6 at different voltages. The inset shows an EL emission photograph of device W6 at 4 V.

Based on the above strategies, high-performance hybrid WOLEDs can be expected. After the overall optimization of the structure parameters, the resulting WOLEDs show very impressive EL performances. The turn-on voltage of the optimized WOLED (device W6) is as low as 2.5 V, as shown in Fig. 4(a), which should be mainly attributed to the “barrier-free” architecture that consists of the bipolar charge transporting ability of mixed fluorescent blue EML, where the charge carriers can be injected directly from the charge transport layer into EML without any obstacles.^6^

As shown in Fig. 4(b) and (c), the device W6 achieves a forward-viewing maximum power efficiency (PE), current efficiency (CE) and external quantum efficiency (EQE) of 72.9 lm W^−1^, 60.3 cd A^−1^ and 18.2% without using light out-coupling technique, which decrease slightly to 61.7 lm W^−1^, 51.1 cd A^−1^ and 16.7% at 100 cd m^−2^ and remain 44.1 lm W^−1^, 45.0 cd A^−1^ and 13.7% at 500 cd m^−2^, respectively. It is worth mentioning that a forward-viewing maximum EQE of 18.2% is achieved at a current density of 0.025 mA cm^−2^, assuming an optical out coupling efficiency of roughly 21%. This corresponds to an IQE close to unity, implying the full conversion of electrically generated excitons into light. The basic device performances are summarized in Table 2, where the performances of some reported hybrid WOLEDs are also given for comparison. It can be seen that our devices exhibit higher efficiencies, at a luminance of 1000 cd m^−2^, EQE, PE and CE are still as high as 12.5%, 37.7 lm W^−1^, and 40.8 cd A^−1^, which should be better results reported so far for hybrid WOLEDs.

**Table 2:** EL performances of some representative and our hybrid WOLEDs

Device	*V*_on_ a (V)	Max/@100 cd m^−2^/@1000 cd m^−2^			CIE^b^ (*x*,*y*)
		CE (cd A^−1^)	PE (lm W^−1^)	EQE (%)	
Ref. 6	2.4	53.5/49.8/42.6	67.2/52.1/33.5	26.6/24.8/21.2	(0.46,0.44)
Ref. 28	2.6	56.1/54.1/47.8	62.9/60.4 /41.7	23.8/23.3/20.1	(0.49,0.41)
Ref. 29	2.9	49.4/—/—	53.5/—/—	19.0/—/—	(0.46, 0.46)
Ref. 30	3.1	—/—/—	51.2/—/38.7	20.8/—/19.6	(0.39, 0.45)
Ref. 31	3.1	45.2/—/40.5	41.7/—/34.3	19.0/—/17.0	(0.43, 0.43)
Ref. 32	3.2	42.5/37.8/—	29.6/25.1/—	15.7/14.0/—	(0.46, 0.43)
Ref. 33	3.3	49.6/—/49.5	40.7/—/37.1	21.1/—/20.0	(0.43, 0.43)
Ref. 34	3.3	55.2/16.6/13.2	49.6/25.7/9.0	20.0/13.2/7.4	(0.38, 0.46)
Ref. 35 c	3.2	45.6/36.2/16.4	35.8/21.6/6.9	16.0/14.1/6.7	(0.34,0.44)
Ref. 36	3.2	56.0/—/48.5	55.5/—/41.5	19.3/—/17.2	(0.33, 0.46)
This work	2.5	60.3/51.1/40.8	72.9/61.7/37.7	18.2/16.7/12.5	(0.40,0.49)

a The turn-on voltage measured at >1 cd m^−2^.

b Measured at 1000 cd m^−2^.

c Represents one all-phosphor devices.

As displayed in Fig. 4(d), device W6 emits good white light with three peaks, respectively, from the blue light of 4P-NPD : Bepp_2_, the green light of Ir(ppy)_2_(acac) and the yellow light of Ir(tptpy)_2_(acac). The Commission Internationale de L'Eclairage (CIE) coordinates are shifted from (0.42, 0.51) to (0.35, 0.46) when the applied voltage is varied from 3 to 7 V, which the homologous luminance increases from 311 cd m^−2^ to 36 400 cd m^−2^. The corresponding correlated colour temperature (CCT) goes from 3862 K to 4950 K. When the luminance is 1000 cd m^−2^, the CCT is around 4000 K very close to the warm white illumination, meeting the requirement of indoor lighting. These performances are comparable to or even higher than those of all phosphor-doped WOLEDs reported before.^25–27^

#### 3.2.3 Detailed analysis on operational mechanism of hybrid WOL-EDs

##### I. Effect of 4P-NPD and Bepp_2_ ratio

**Fig. 5:**
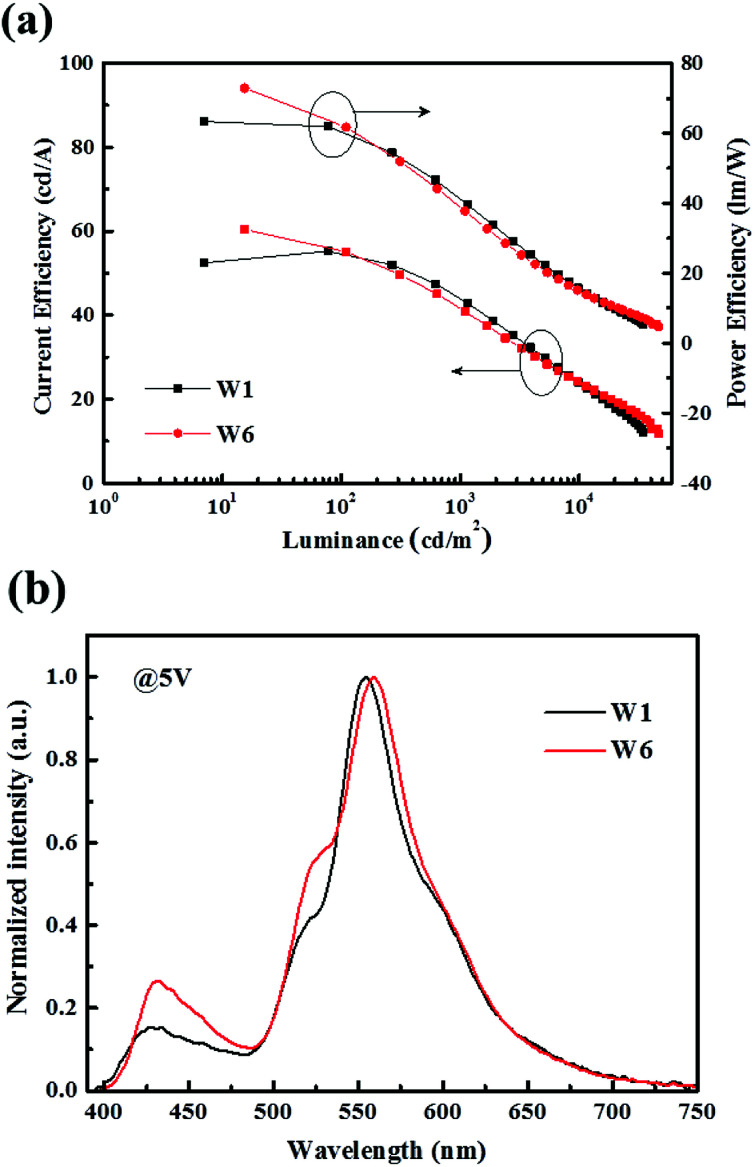
(a) CE–PE–L characteristics of device W1 and W6. (b) Normalized EL spectra of device W1 and W6 at 5 V.

From the previous section, we know that the optimum ratio of 4P-NPD and Bepp_2_ in blue OLEDs is confirmed to be 2 : 1. In order to explore the ratio influence in the hybrid WOLEDs, we have made a comparison device with structure of ITO/HAT-CN (15 nm)/TAPC : HAT-CN (4/2, 95 nm)/TAPC (40 nm)/4P-NPD:Ir(tptpy)_2_(acac) (4%, 5 nm)/4P-NPD : Bepp_2_ (*X*, 13 nm)/Bepp_2_ (2 nm)/Ir(ppy)_2_(acac) (0.4 nm)/Bepp_2_ (3 nm)/Bepp_2_:Liq (4.5%, 50 nm)/Liq (1.25 nm)/Al, *X* = 1 : 1, refer to device W1. As depicted in Fig. 5(a), the efficiency of two devices is different under low voltage. The efficiency of device W6 is higher than that of device W1. Although there is little difference in their efficiency at high voltage, as portrayed in Fig. 5(b), where the normalized EL spectra of device W1 and W6 are given, device W1 emits lower blue and green light intensity with respect to device W6, which should be necessary for a good white light emission.

##### II. Emission mechanism of phosphors

**Fig. 6:**
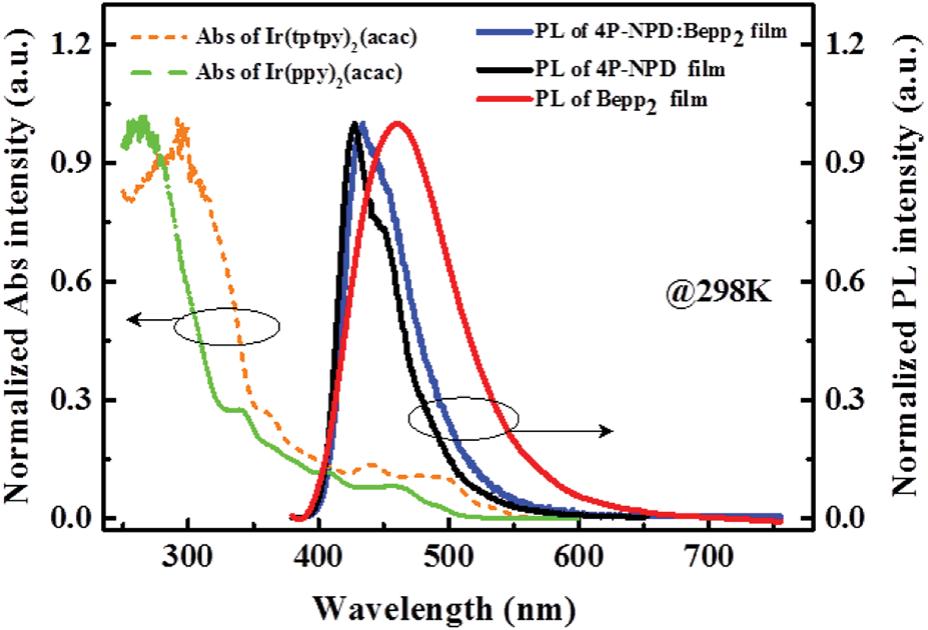
Normalized absorption spectra of Ir(ppy)_2_(acac) and Ir(tptpy)_2_(acac) measured in CH_2_Cl_2_ solvent as well as photoluminescence spectra of pure 4P-NPD, pure Bepp_2_, and 4P-NPD : Bepp_2_ (2 : 1) mixture solid films.

As shown in Fig. 6, there is a large overlap between the absorption spectra of Ir(ppy)_2_(acac), Ir(tptpy)_2_(acac) and the emission spectra of 4P-NPD, Bepp_2_ and 4P-NPD : Bepp_2_. This means a large spectra overlap between the fluorescence band of the ligand-centered singlet (^1^LC) states in 4P-NPD and the absorption bands of both metal ligand charge transfer singlet (^1^MLCT) and triplet (^3^MLCT) states in Ir(ppy)_2_(acac) and Ir(tptpy)_2_(acac). As a result, the lowest singlet-excited states (S_1_) in 4P-NPD can be energetically transferred to either the lowest triplet-excited states (T_1_) or the S_1_ (which then transits to the T_1_*via* rapid intersystem crossing (ISC) for the strong spin–orbit coupling) in Ir(ppy)_2_(acac) and Ir(tptpy)_2_(acac) by Förster energy transfer (Coulomb interaction).^23,37^ Furthermore, according to the previous reports,^15,16,38^ the T_1_ values of 4P-NPD and Bepp_2_ are 2.3 eV and 2.6 eV respectively, and the T_1_ values of Ir(ppy)_2_(acac) and Ir(tptpy)_2_(acac) are 2.4 eV and 2.2 eV, thus ensuring the efficient T_1_ (Bepp_2_) → T_1_ (Ir(ppy)_2_(acac) and Ir(tptpy)_2_(acac)) transition and T_1_ (4P-NPD) → T_1_ (Ir(tptpy)_2_(acac)) transition through a Dexter-type (electron-exchange) interaction because of their T_1_ energy level difference. In addition, the PL spectra show that 4P-NPD : Bepp_2_ film does not show a long wavelength emission, excluding the possibility of exciplex formation in our material system. As shown in Fig. 6, the blue emission of 4P-NPD : Bepp_2_ blend film is obviously from the co-emission of 4P-NPD and Bepp_2_, which is the special point in our devices.

**Fig. 7:**
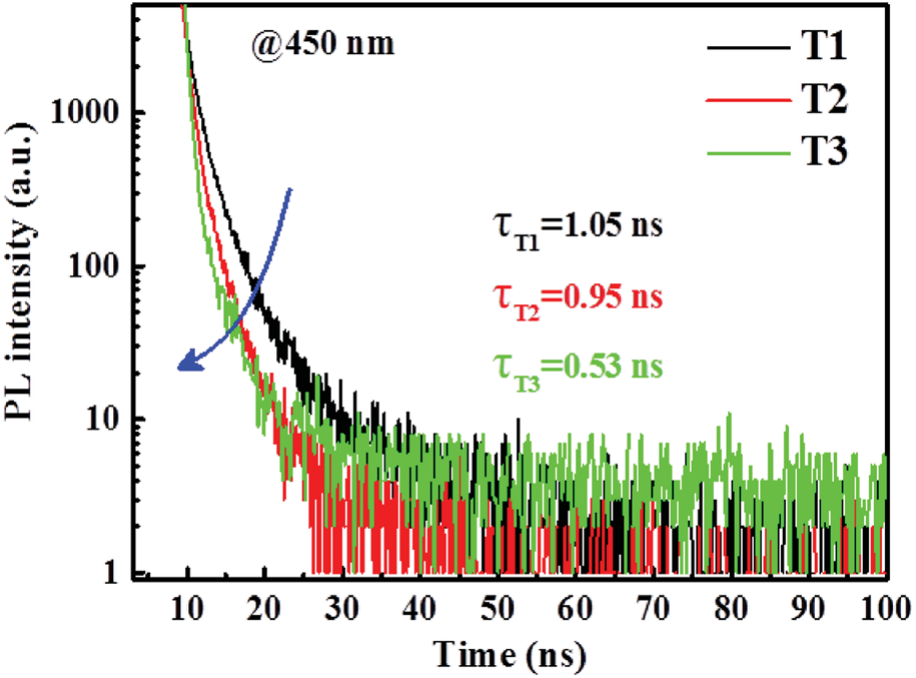
PL transient decay curves of three different films tested at 450 nm (PL emission peak of Bepp_2_). Related film structures are as follows: film T_1_: 4P-NPD (5 nm)/4P-NPD : Bepp_2_ (2 : 1, 13 nm)/Bepp_2_ (2 nm), film T_2_: 4P-NPD:Ir(tptpy)_2_(acac) (4%, 5 nm)/4P-NPD : Bepp_2_ (2 : 1, 13 nm)/Bepp_2_ (2 nm), film T_3_: 4P-NPD (5 nm)/4P-NPD : Bepp_2_ (2 : 1, 13 nm)/Bepp_2_ (2 nm)/Ir(ppy)_2_(acac) (0.4 nm).

In order to further confirm the Förster energy transfer between fluorophors and phosphors, we tested the PL transient decay lifetime of blue fluorescence in three different films. As shown in Fig. 7, where the PL transient decay curves of the 4P-NPD : Bepp_2_ mixed films with or without phosphorescent layer are given. Clearly, the blue lifetime is obviously decreased from 1.05 ns of film T_1_ to 0.95 ns of film T_2_, and to 0.53 ns of film T_3_. As known to all, under PL excitation, the formation of excitons is only singlet states. The lifetime reduction of the blue emission when adding phosphorescent layer means the Förster energy transfer from the singlet states of 4P-NPD and Bepp_2_ to the triplet states of phosphors Ir(ppy)_2_(acac) and Ir(tptpy)_2_(acac) and the green Ir(ppy)_2_(acac) molecules obtain more energies.

**Fig. 8:**
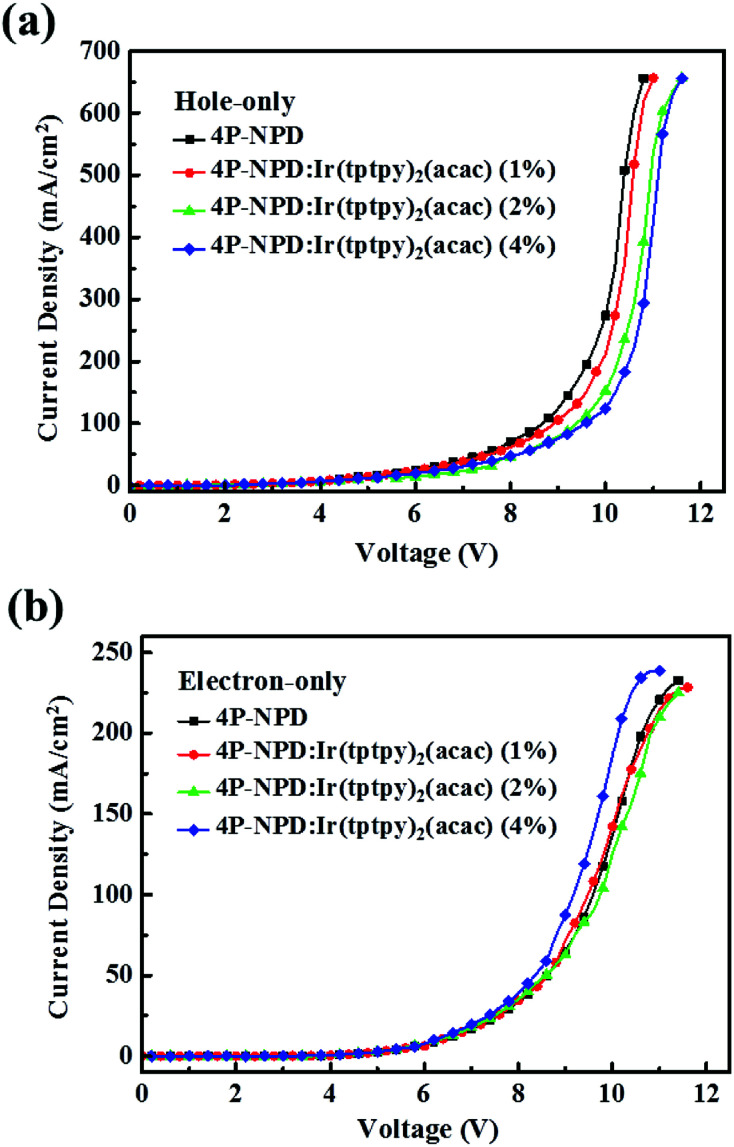
Current density–voltage (*J*–*V*) characteristics of (a) hole-only devices: ITO/HAT-CN (15 nm)/TAPC : HAT-CN (4/2, 95 nm)/TAPC (40 nm)/4P-NPD:Ir(tptpy)_2_(acac) (*X* = 0,1,2,4%, 5 nm)/4P-NPD : Bepp_2_ (2 : 1, 13 nm)/TAPC (40 nm)/TAPC : HAT-CN (4/2, 95 nm)/HAT-CN (15 nm)/Al, and (b) electron-only devices: ITO/Liq (1.25 nm)/Bepp_2_ (50 nm)/4P-NPD:Ir(tptpy)_2_(acac) (*X* = 0,1,2,4%, 5 nm)/4P-NPD : Bepp_2_ (2 : 1, 13 nm)/Bepp_2_ (40 nm)/Liq (1.25 nm)/Al.

It is widely believed that the emitting light mechanisms in doped OLEDs include the direct recombination of carriers and energy transfer. In order to further explore the mechanism of yellow light in our hybrid WOLEDs, the hole-only and electron-only devices were fabricated. Their *J*–*V* characteristics are shown in Fig. 8. It can be seen that Ir(tptpy)_2_(acac) has a little trapping effect on the holes, whereas the capture of electrons is almost negligible. This also means that the yellow emission is indeed energy transfer processes, and mainly the Dexter energy transfer from the triplet states of 4P-NPD and Bepp_2_ to the triplet states of Ir(tptpy)_2_(acac), whereas the Förster energy transfer from the singlet states of 4P-NPD to the triplet states of Ir(tptpy)_2_(acac) is almost negligible. To verify this assumption, we made the devices with different 4P-NPD : Bepp_2_ thicknesses according to the different diffusion distances of singlet and triplet excitons.

**Fig. 9:**
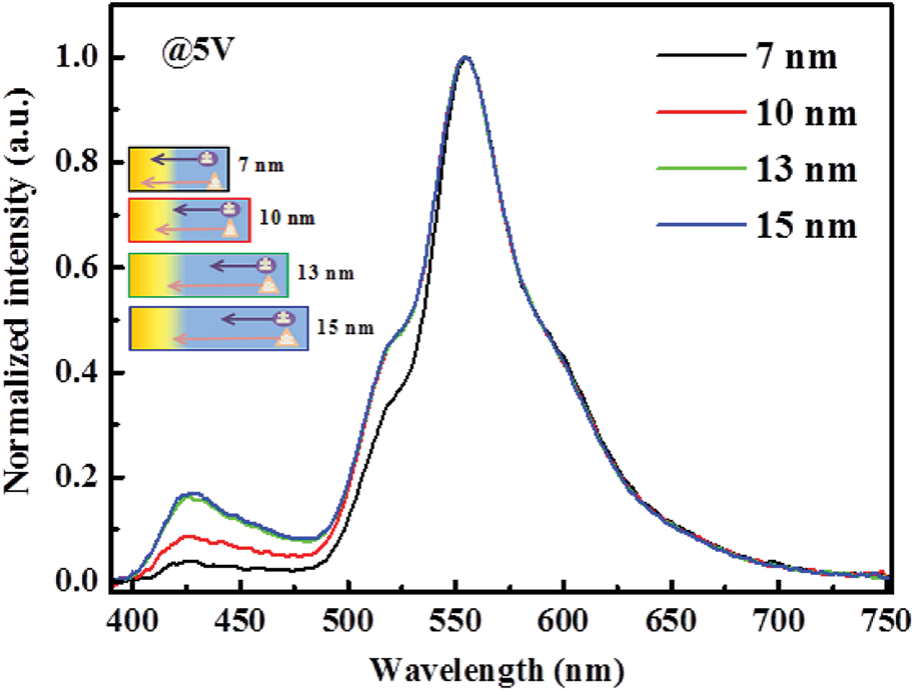
Normalized EL spectra of devices with different blue EML thicknesses of 5, 10, 13 and 15 nm. The device structure is ITO/HAT-CN (15 nm)/TAPC : HAT-CN (4/2, 95 nm)/TAPC (40 nm)/4P-NPD:Ir(tptpy)_2_(acac) (4%, 5 nm)/4P-NPD : Bepp_2_ (2 : 1, *X* nm)/Bepp_2_ (2 nm)/Ir(ppy)_2_(acac) (0.4 nm)/Bepp_2_ (3 nm)/Bepp_2_:Liq (4.5%, 50 nm)/Liq (1.25 nm)/Al. The inset marks the different diffusion lengths of S_1_ and T_1_ excitons.

As shown in Fig. 9, we can find that the blue light intensity becomes very weak when the thickness is less than 7 nm, indicating the strong Förster energy transfer from 4P-NPD and Ir(tptpy)_2_(acac). When the blue EML thickness is over 13 nm, the blue intensity varies with the thickness, indicating that the Förster energy transfer between 4P-NPD and Ir(tptpy)_2_(acac) is negligible. Therefore, it is concluded that the yellow light emission in our hybrid WOLEDs is mainly attributed to the Dexter energy transfer from 4P-NPD and Bepp_2_ to Ir(tptpy)_2_(acac).

**Fig. 10:**
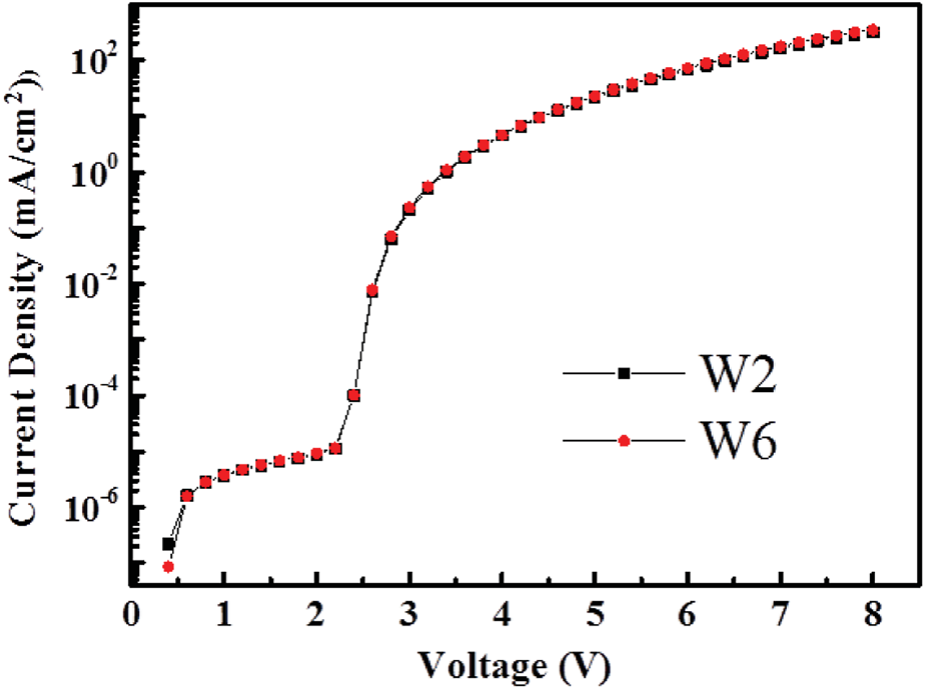
*J*–*V* characteristics of device W2 and W6.

For the emission of the ultra-thin phosphor, there are a lot of literatures about WOLEDs that prove to be only energy transfer.^12,39^ In order to demonstrate the emission process of the ultra-thin green phosphor in our hybrid WOLEDs, we have made a device W2 without the ultra-thin green phosphor for comparison. The device W2 structure is ITO/HAT-CN (15 nm)/TAPC : HAT-CN (4/2, 95 nm)/TAPC (40 nm)/4P-NPD:Ir(tptpy)_2_(acac) (4%, 5 nm)/4P-NPD : Bepp_2_ (2 : 1, 13 nm)/Bepp_2_ (5 nm)/Bepp_2_:Liq (4.5%, 50 nm)/Liq (1.25 nm)/Al. It can be seen from the *J*–*V* characteristics of device W2 and W6 in Fig. 10 that there is no obvious differences between two devices, excluding the existence of charge carrier trapping on green phosphor. This also means that the green emission from Ir(ppy)_2_(acac) molecules is also energy transfer.

#### 3.2.4 Role of ultra-thin green phosphor layer

As we see, the incorporated 0.4 nm green ultrathin emitting layer also plays an important role in improving efficiency and efficiency roll-off. To examine its role, we made the device W2 without the ultrathin green EML as contrast to the optimism device W6. The device W2(Y/B) configuration is as follows: ITO/HAT-CN (15 nm)/TAPC : HAT-CN (4/2, 95 nm)/TAPC (40 nm)/4P-NPD:Ir(tptpy)_2_(acac) (4%, 5 nm)/4P-NPD : Bepp_2_ (2 : 1, 13 nm)/Bepp_2_ (5 nm)/Bepp_2_:Liq (4.5%, 50 nm)/Liq (1.25 nm)/Al.

**Fig. 11:**
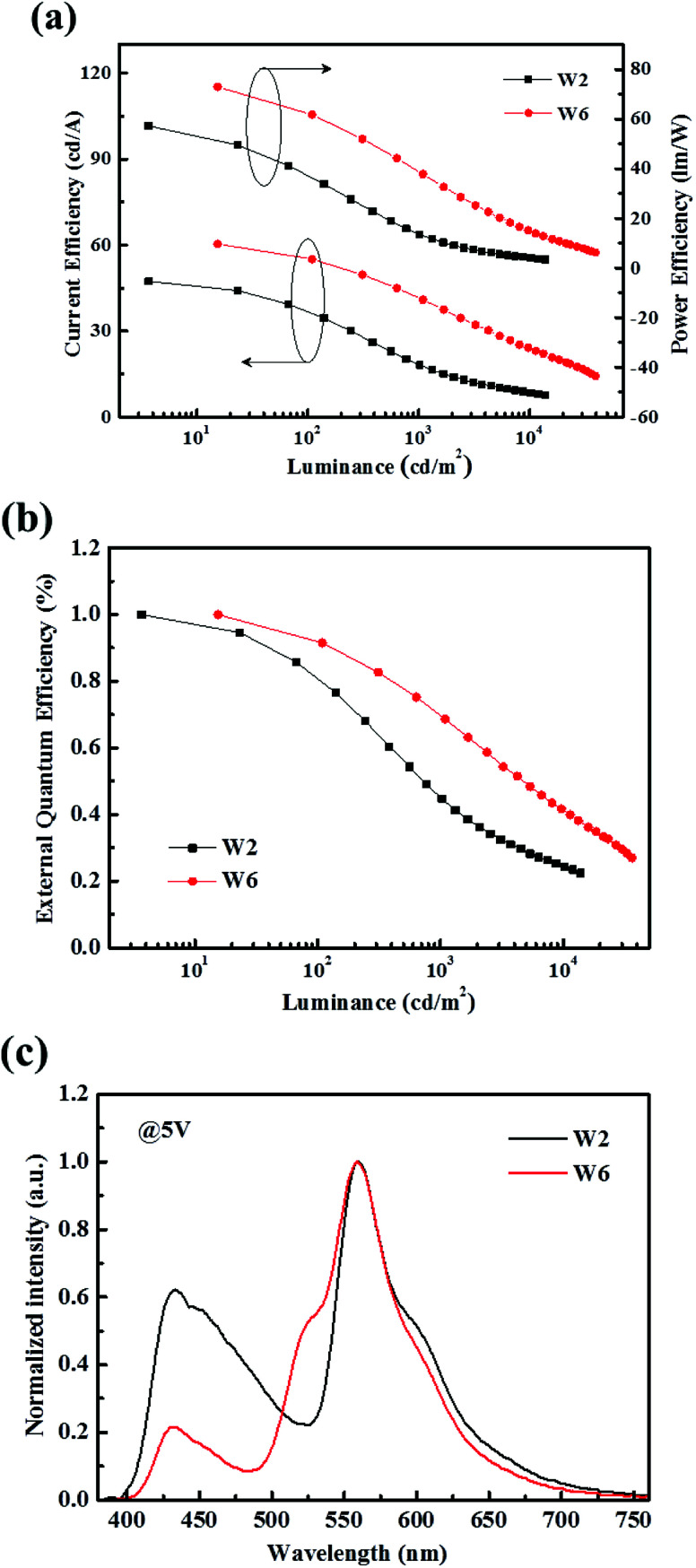
(a) CE–PE–L characteristics of device W2 and W6. (b) Normalized EQE-L characteristics of device W2 and W6. (c) Normalized EL spectra of device W2 and W6.

As displayed in Fig. 11(a) and (b), we can find that the introduction of the ultrathin green EML not only greatly enhances the efficiency, but also significantly improves the efficiency roll-off at high luminance. This can also be explained from the EL spectra shown in Fig. 11(c), the inserted ultrathin green EML absorbed partial exciton energies in blue EML, resulting in the green emission, and the reduction of the blue emission in device W6. This process should be very favor in reducing the quenching caused by exciton aggregation. For comparison purposes and clarity, the EL performances of devices W1, W2 and W6 are shown in Table S1.†

#### 3.2.5 Quenching mechanism in device W6

As we know, the efficiency roll-off is a common problem in OLEDs, which may limit their implementation at high luminance. Generally, triplet–triplet annihilation (TTA), triplet-polaron quenching (TPQ) and field-induced quenching are possible quenching processes in OLEDs.^40^ For TTA model, as reported by Baldo *et al.*,^40^ the EQE (*η*) dependence of current density (*J*) can be expressed as follows,1
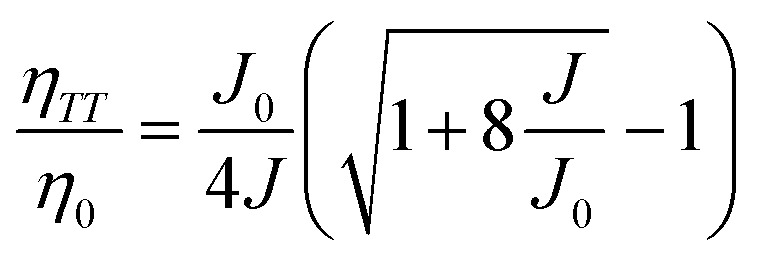


**Fig. 12:**
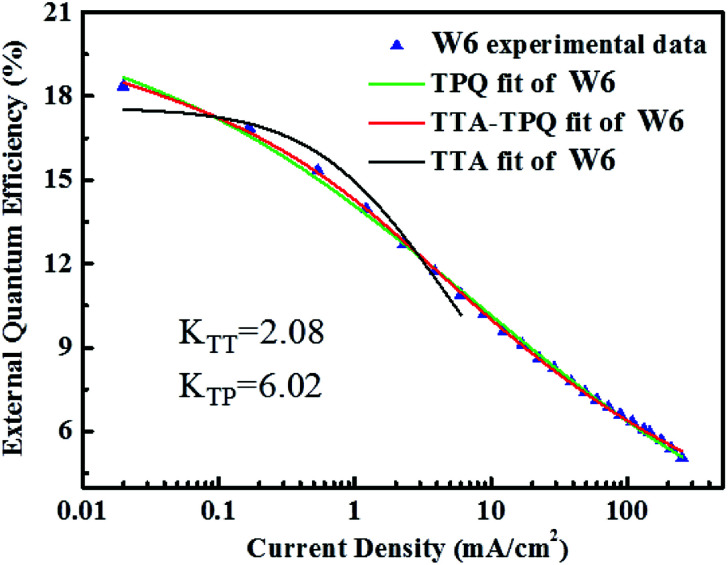
EQE-J dependence of device W6. The green line refers to the TPQ fitting curve and the red line refers to the TTA-TPQ fitting curve. The black line refers to the TTA fitting curve.

While for TPQ model, the expression can be described by S. Reineke *et al.*,^41^2
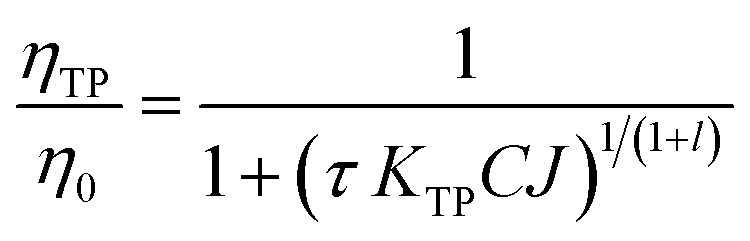
Here *η*_0_ is the EQE without the triplet exciton quenching, and *J*_0_ is the critical current density at *η* = *η*_0_/2, *τ* is the decay lifetime of the triplet, *C* is a constant related to the parameters such as the dielectric constant and carrier mobility, *K*_TP_ is the TPQ rate constant, 1 is taken to be unity. The unified model proposed by S. Reineke *et al.*^41^ incorporates the TTA and TPQ quenching mechanisms, which is given as follows,3
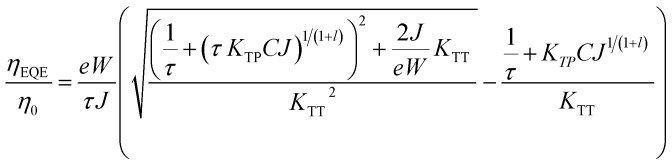
where *W* represents the width of exciton recombination zone, *K*_TT_ is the TTA rate constant, the other parameters are the same as the mentioned above. The EQE of devices W6 as a function of current density and the fitting according to the unified model (red line) integrating the TTA with TPQ mechanism are shown in Fig. 12. It is demonstrated that our experimental data can be well fitted by TTA-TPQ model.

As shown in Fig. 12, our experimental data can be well fitted by TPQ model in high brightness, and there is no obvious difference between TPQ model and TTA-TPQ model, which indicating that the TPQ process is the main reason for the efficiency roll-off at high luminance in our devices. *K*_TT_(≈2.08) is much lower than *K*_TP_(≈6.02), which indicates that the efficiency roll-off at high luminance in device W6 is mainly due to the triplet-polaron quenching. The failure of TTA model fitting implies that the wide exciton recombination zone (as shown in Fig. 2) conduces the decrease of triplet exciton concentration, thus reducing the TTA effect and finally resulting in the reduced efficiency roll-off. The good TPQ model fitting implies that there exist unbalanced charge injection and transport in our devices, leading to severe charge accumulation, thus quenching emissive excitons.

## 4. Conclusions

We have successfully achieved high-performance hybrid WOLEDs by simply and effective exciton regulation emissive layer structure. It can be seen that the strategic introduction of an ultra-thin green phosphor EML in proper location in ETL not only greatly enhances the efficiency, but also significantly improves the efficiency roll-off at high luminance, which is attributed to the efficient regulation in the emissive excitons.

This strategy has rendered a record maximum forward viewing external quantum efficiency and power efficiency of 18.2% and 72.9 lm W^−1^, and they are as high as 16.7% and 61.7 lm W^−1^ at 100 cd m^−2^, and 12.5% and 37.7 lm W^−1^ at 1000 cd m^−2^, respectively, in our hybrid WOLEDs without any out-coupling assistance. Such productive results indicate a promising way to promote the development of high-performance hybrid WOLEDs by simple structure, which is very valuable for the further design of high-performance WOLEDs in the future.

## Conflicts of interest

There are no conflicts to declare.

## Supplementary Material

RA-008-C8RA08753A-s001
