# Supplementary material for: High efficiency hybrid white organic light-emitting diodes based on a simple and efficient exciton regulation emissive layer structure
Source: RSC Adv. 2018 Dec 6;8(71):40883–93. doi: 10.1039/c8ra08753a (PMC9091617; doi:10.1039/c8ra08753a)
Supplement: RA-008-C8RA08753A-s001 [file RA-008-C8RA08753A-s001.pdf]

Electronic Supplementary Material (ESI) for RSC Advances.
This journal is © The Royal Society of Chemistry 2018



# Electronic Supplementary Information

## High Efficiency Hybrid White Organic Light-Emitting Diodes based on a Simple and Efficient Exciton Regulation Emissive Layer Structure

Yuwen Chen, Shian Ying, Qian Sun, Yanfeng Dai, Xianfeng Qiao, Dezhi Yang, Jiangshan Chen and Dongge Ma*

*Institute of Polymer Optoelectronic Materials and Devices, State Key Laboratory of Luminescent Materials and Devices, South China University of Technology, Guangzhou, 510640, People's Republic of China*

E-mail: msdgma@scut.edu.cn

# Contents

## Figures

**Figure S1**. Schematic structure of designed Blue OLED

**Figure S2**. The molecular structures of organic materials used for OLED in this work.

**Table S1**. Summary of EL performances of the resulting WOLEDs.

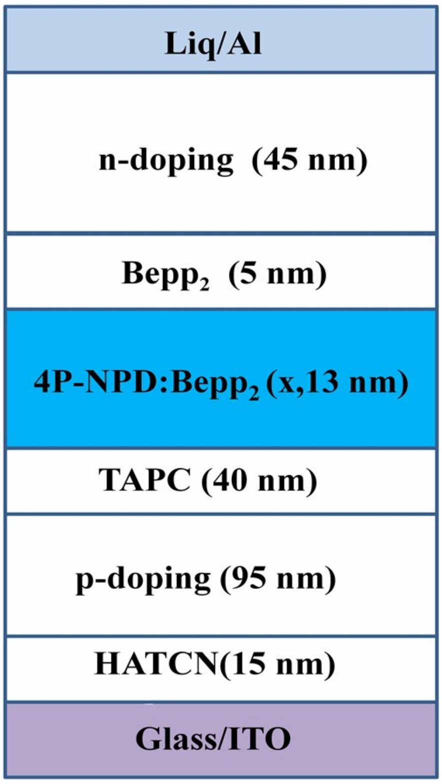


**Figure S1.**Schematic structure of designed Blue OLED

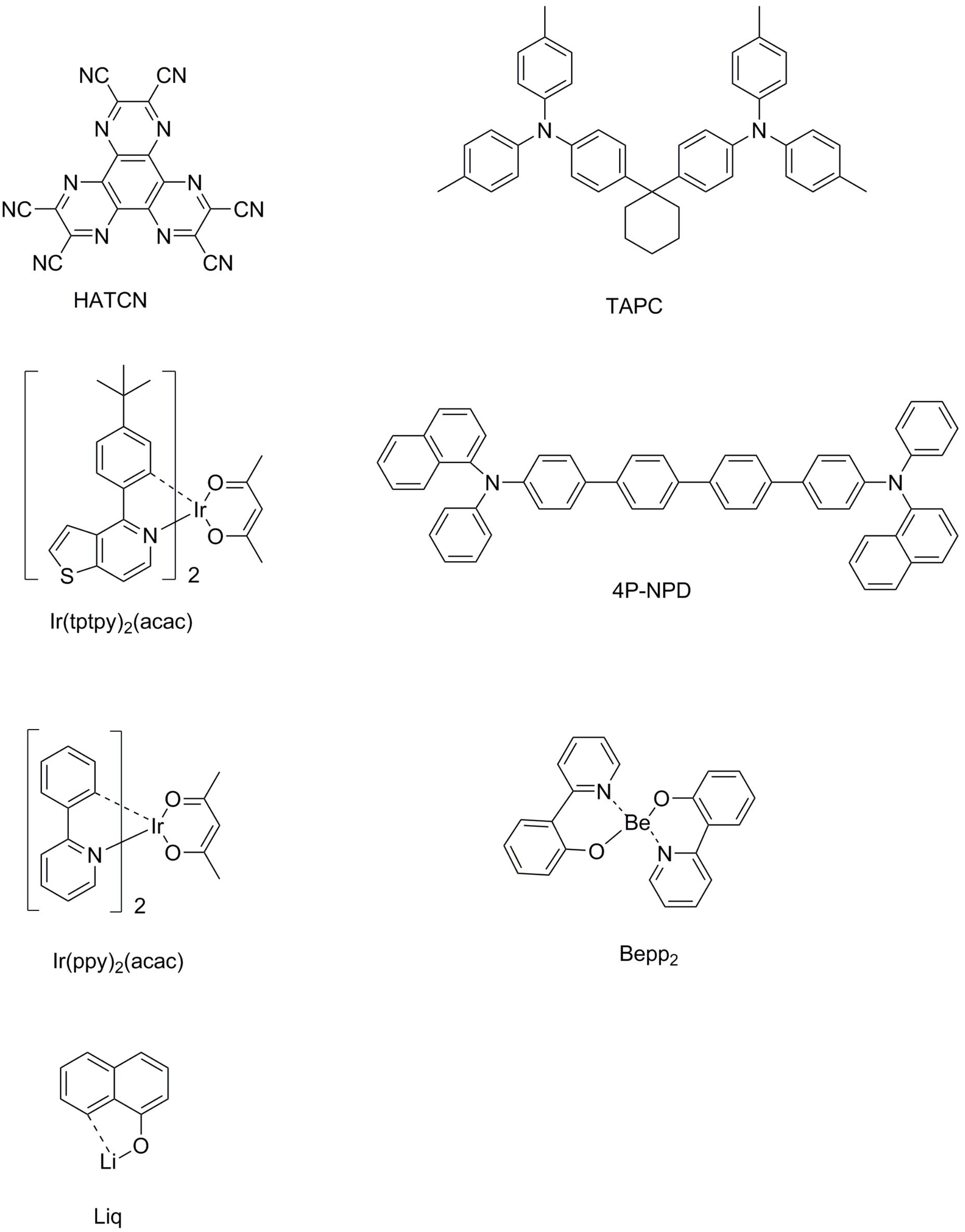


**Figure S2.** The molecular structures of organic materials used for OLED in this work.

**W1 structure**: ITO/HAT-CN (15 nm)/TAPC: HAT-CN (4/2, 95 nm)/TAPC (40 nm)/4P-NPD: Ir(tptpy)$_2$(acac) (4%, 5 nm)/4P-NPD: Bepp$_2$ (1: 1, 13 nm)/Bepp$_2$ (2 nm)/Ir(ppy)$_2$(acac) (0.4 nm)/Bepp$_2$ (3 nm)/Bepp$_2$: Liq (4.5%， 50 nm)/Liq ( 1.25 nm)/Al,

**W2 structure**: ITO/HAT-CN (15 nm)/TAPC: HAT-CN (4/2, 95 nm)/TAPC (40 nm)/4P-NPD: Ir(tptpy)$_2$(acac) (4%, 5 nm)/4P-NPD: Bepp$_2$ (2: 1, 13 nm)/Bepp$_2$ (5nm)/Bepp$_2$: Liq (4.5%, 50 nm)/Liq ( 1.25 nm)/Al.

**W6 structure**: ITO/HATCN (15 nm)/TAPC: HATCN (4/2, 95 nm)/TAPC (40 nm)/4P-NPD: Ir(tptpy)$_2$(acac) (4%, 5 nm)/4P-NPD: Bepp$_2$ (2: 1, 13 nm)/Bepp$_2$ (2 nm)/Ir(ppy)$_2$(acac) (0.4 nm)/Bepp$_2$ (3 nm)/Bepp$_2$: Liq (4.5%, 50 nm)/Liq (1.25 nm)/Al.

**Table S1** Summary of EL performances of the resulting WOLEDs.

| Device | $V_{on}$[a] (V) | Max/@100 cd $m^{-2}$/@1000 cd $m^{-2}$ | | | CRI[b] | CIE[b] (x,y) |
|---|---|---|---|---|---|---|
| | | CE (cd $A^{-1}$) | PE (lm $W^{-1}$) | EQE (%) | | |
| W1 | 2.6 | 58.3/56.7/48.7 | 65.5/59.3/44.9 | 16.7/13.5/11.0 | 42 | (0.43,0.52) |
| W2 | 2.6 | 47.3/34.5/18.2 | 57.2/33.9/13.5 | 15.8/12.1/13.5 | 51 | (0.36,0.37) |
| W6 | 2.6 | 60.3/40.8/28.3 | 72.9/37.7/20.2 | 18.2/12.5/8.8 | 46 | (0.40,0.49) |

[a] Turn-on voltage estimated at a brightness of >1cd $m^{-2}$. [b] Emission peak and the corresponding CRI and CIE coordinates obtained at a voltage of 4 V.

**Table S1.** Summary of EL performances of the resulting WOLEDs.
